# Impact of climate change on larch budmoth cyclic outbreaks

**DOI:** 10.1038/srep27845

**Published:** 2016-06-13

**Authors:** Sudharsana V. Iyengar, Janaki Balakrishnan, Jürgen Kurths

**Affiliations:** 1 School of Physics, University of Hyderabad, Central Univ. PO, Gachi Bowli, Hyderabad 500 046, India; 2 School of Natural Sciences & Engineering, National Institute of Advanced Studies (N.I.A.S.), Indian Institute of Science Campus, Bangalore - 560012, India; 3 Potsdam Institute for Climate Impact Research, PO Box 601203, Potsdam 14412, Germany

## Abstract

Periodic outbreaks of the larch budmoth *Zeiraphera diniana* population (and the massive forest defoliation they engender) have been recorded in the Alps over the centuries and are known for their remarkable regularity. But these have been conspicuously absent since 1981. On the other hand, budmoth outbreaks have been historically unknown in the larches of the Carpathian Tatra mountains. To resolve this puzzle, we propose here a model which includes the influence of climate and explains both the 8–9 year periodicity in the budmoth cycle and the variations from this, as well as the absence of cycles. We successfully capture the observed trend of relative frequencies of outbreaks, reproducing the dominant periodicities seen. We contend that the apparent collapse of the cycle in 1981 is due to changing climatic conditions following a tipping point and propose the recurrence of the cycle with a changed periodicity of 40 years – the next outbreak could occur in 2021. Our model also predicts longer cycles.

Tree-ring analysis of larch trees in the Alpine region have shown 123 outbreaks over the past 1200 years. While the predominant periodicity found is of around 9 years, an 8 year cycle has also been observed, and (less frequently) a 10 year cycle, with other periodic outbreaks occurring a fewer number of times^1^. The last outbreak occurred in 1981 after which the cycles have collapsed^2^. Experiments reported in ref. 3 revealed changes in periodicity when the densities of the budmoths were altered by manual removal or spraying of pesticides. Such outbreaks are not seen in the Tatra mountains in the Carpathians. Trees in the Tatra mountain range are at much lower altitudes and are hence exposed to much warmer conditions in comparison to their Alpine counterparts. The presence of a constant cloud cover in the Tatras also exposes these larches to less sunshine, and they are sparser – another distinguishing feature between the Alpine and Tatra larches^4^. The absence of budmoth outbreaks in the Tatra region^4^ as opposed to that in the Alps has been a puzzle thus far and a challenge for modelling the ecological system.

In this contribution we propose a mathematical framework that enables us to capture all the above mentioned facts along with the observed relative frequencies of occurrence of the various periodicities as well as the absence of expected cycles. Additionally, our model predicts future possible cycles.

Wasteful feeding by budmoth larvae on larch foliage leading to a scorched appearance of the entire landscape, has prompted several investigations of the population cycles of this insect pest (see for instance ref. 5). Previous studies on larch budmoth (LBM) population cycles have established the presence of a third trophic level – parasitoids which prey upon budmoth larvae^6^,^7^,^8^,^9^. The population densities of the budmoth, the parasitoids preying upon them and needle lengths of the larch^10^ are all known to show periodic cycles which are mutually synchronized. This tritrophic system was first modelled by Turchin^8^ (see Methods).

On an average, larch needles grow upto 30 mm in length, lengthier needles being an indicator of good health. The current health of the plant which depends upon its nutrient supply and its previous state after the last budmoth infestation is hence captured well through a dimensionless Plant Quality Index (PQI) which is directly related to the needle length^9^ (see Methods). Turchin’s model^8^ had tightly tuned parameters, adjusted to match the observed 9 year budmoth cycle.

In ref. 11 we considered this tritrophic system using the dimensionless scaling introduced in ref. 12; however the decay of foliage was no longer represented by a constant, but rather by a density-dependent function of the PQI.

In the present work, we substantially generalize our model to incorporate, for the first time, factors which relate to environmental and climatic conditions.

## Model for the Tritrophic System with Climate Parameters

Our description of the tritrophic system is represented in the following model (see Methods):


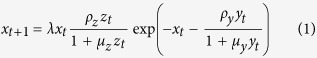



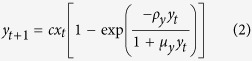



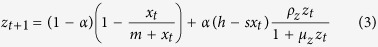


where 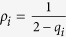, 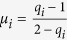, *x*_*t*_ and *y*_*t*_ denote respectively the population densities of budmoth and parasitoid in dimensionless form and *z*_*t*_ the PQI at time *t*. Other factors influencing larch growth are modelled thus: the intrinsic growth rate of the budmoth is denoted by *λ*, *α* denotes plant-vulnerability; *h* is a climate/environmental factor and *s* the budmoth-larch interaction strength which depends upon climatic conditions. The other system parameters are the efficiency *c* of the parasitoid in parasitising the budmoth, the budmoth’s infestation efficiency *m*, *q*_*z*_ which is related to its intraspecific competition and *q*_*y*_ which is related to the parasitoid wasting time.

Equation 3 (that governs the PQI) now has three contributions therein – one from the evolution of infestation-free leaves (term without climate factors), another from the regeneration (under favourable climatic conditions) of previously damaged larch needles (term containing *h*); and third, a density dependent interaction term representing current budmoth infestation (with factor *s*).

Incorporating climatic effects influencing this system is especially important in correctly capturing its evolution in a model. In particular, warm weather immediately after snow thaw and direct exposure to sunlight helps larch foliage thrive during the vegetation period^13^. Growth is enhanced by an early, warm springtime, but a late, cool spring retards growth^10^. Short, dry summers and very cold winters are conditions favourable to the larch. Good precipitation is beneficial for both larch and budmoth, although continuous cloud cover hampers larch growth^4^. Increased overall temperatures in the active vegetation period and increased frost do not appear to favour larch growth^14^. Our model, with eight parameters, is the most versatile one available to analyze the system and includes the effect of climate. In general, large values of *h* and *s* can be taken to correspond to favourable environmental conditions, and low values to adverse conditions. We can, for example, interpret *h* as a measure of precipitation, and *s* as an inverse temperature. If winter temperatures are high, it leads to high egg mortality and hence, low budmoth populations. This important effect of temperature is incorporated in our model through the parameter *s*. Because of the complex relationship between various parameters in the system, a strict interpretation of *all* high *h*, *s* values being uniformly favourable may not, however, be feasible.

Our model incorporates *q*-deformation in the equations, as the tritrophic system’s population cycles bear memory of previous years’ growth of the larch. There is a strong and complex interplay between the parasitoids living off budmoth larvae, the plant quality index measuring the health of the larch, and the larch budmoth. This memory makes the system non-ergodic, since all possible states are no longer equiprobable and certain events may possibly be favoured over others. The probability distribution is now skewed, since the organisms’ behaviour, led by different kinds of signals received at different times^15^,^16^,^17^,^18^, causes a suppression of some probabilities while enhancing others. Owing to the variety of factors contributing to the information content in a biological system at any instant of time, with memory of previous configurations being retained in the system, a Boltzmann-Gibbs distribution that attributes equal probabilities to all situations is no longer applicable and fails. Such systems as well as those with long range interactions and strong correlations are better explained with Tsallis probability distribution^19^ which introduces a parameter *q* that allows, depending upon its value, some probabilities to be suppressed and some to be enhanced. Thus the *q*-value would indicate how the system’s behaviour is manifested — whether through rare events or through common events. Tsallis’ non-extensive statistical mechanics expresses naturally through *q*-deformed numbers, with *q* ≠ 1, the standard Boltzmann-Gibbs distribution being recovered in the limit *q* → 1.

The functional response functions in the tritrophic model which generate hyperbolic response naturally have the form of Jackson’s *q*-deformed numbers^20^ used by Tsallis: 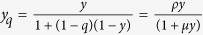 with *q* ≠ 1 — this choice avoids overcounting of individuals (see Methods, eqn. (7)). Our model incorporating *q*-deformation of numbers (corresponding to the choice *q*_*y*_ ≠ 1, *q*_*z*_ ≠ 1 and *q*_*x*_ = 1) is therefore a better and natural candidate to model the tritrophic system.

## Results

**Figure 1:**
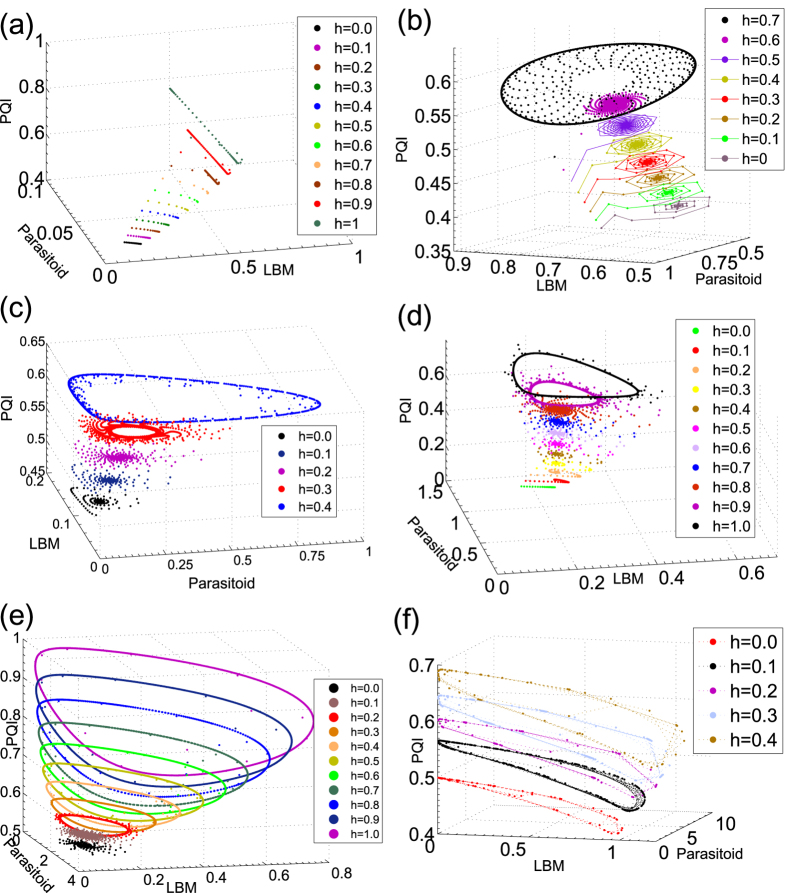
Limit cycles in favorable (large *h* values) and unfavorable (low *h* values) climatic conditions and different intrinsic growth rates *λ* of the budmoth. For very good climatic conditions (large *h* values) limit cycles are born at very low values of growth rate *λ*, while for these to occur in unfavourable conditions, a much higher *λ* is required. The amplitude of the cycle is larger for a given *λ* when *h* is high. For instance, (**a,b**) could well represent the Tatra region, with low *c* values (low parasitoid efficiency/large intra-specific competition among budmoths): limit cycles appear only for higher values of *h* and *λ*. (**c–f**) could represent the Alpine region well: with large *c*, and limit cycles present even for low *h* and *λ*. (**a**) *c* = 2, *λ* = 2, (**b**) *c* = 2, *λ* = 6.5, (**c**) *c* = 12, *λ* = 2, (**d**) *c* = 6, *λ* = 2, (**e**) *c* = 6, *λ* = 3, (**f**) *c* = 12, *λ* = 5. Other parameters (for (**a–f**)): *α* = 0.5, *m* = 13, *q*_*y*_ = 1.13, *q*_*z*_ = 1.34.

The general behaviour is expressed in terms of equilibria: stationary states, periodic solutions, etc. depending upon parameter values. Our model has several equilibria (x^*^, y^*^, z^*^) (see Methods): (i) the stable state of the uninfested larch, (ii) parasitoid-free states and (iii) non-trivial equilibria which can also support periodic behaviour (stable limit cycles) — these produce the observed cycles in the budmoth and parasitoid populations and the needle lengths and are depicted in Fig. 1.

Figure 1(a,b) are reminiscent of the Tatra region where larches grow sparser than in the Alps. This leads to high intraspecific competition between the budmoths, or equivalently, low values for *c*. Cycles could then be sustained only when climatic conditions are very favourable for growth of the larches (large *h* values) and when the budmoth intrinsic growth rate *λ* is very high (see Fig. 1(a,b)). The Tatras which are under cloud cover most of the time, receiving lesser sunshine than the Alps, would be characterized by low *h* values and hence would never have cyclic outreaks. Also *λ*, known to be strongly influenced by the plant quality index^9^ appears to be a distinguishing parameter beteeen the Alps and the Tatras We note that the other parameters are species-specific and can be changed only by long term factors like evolution (or some very drastic change that permanantly changes everything, such as forest fires or some other catastrophe). We have assumed a slow climate change which began towards the end of the last century; our assumption is also consistent with the observations.

**Figure 2:**
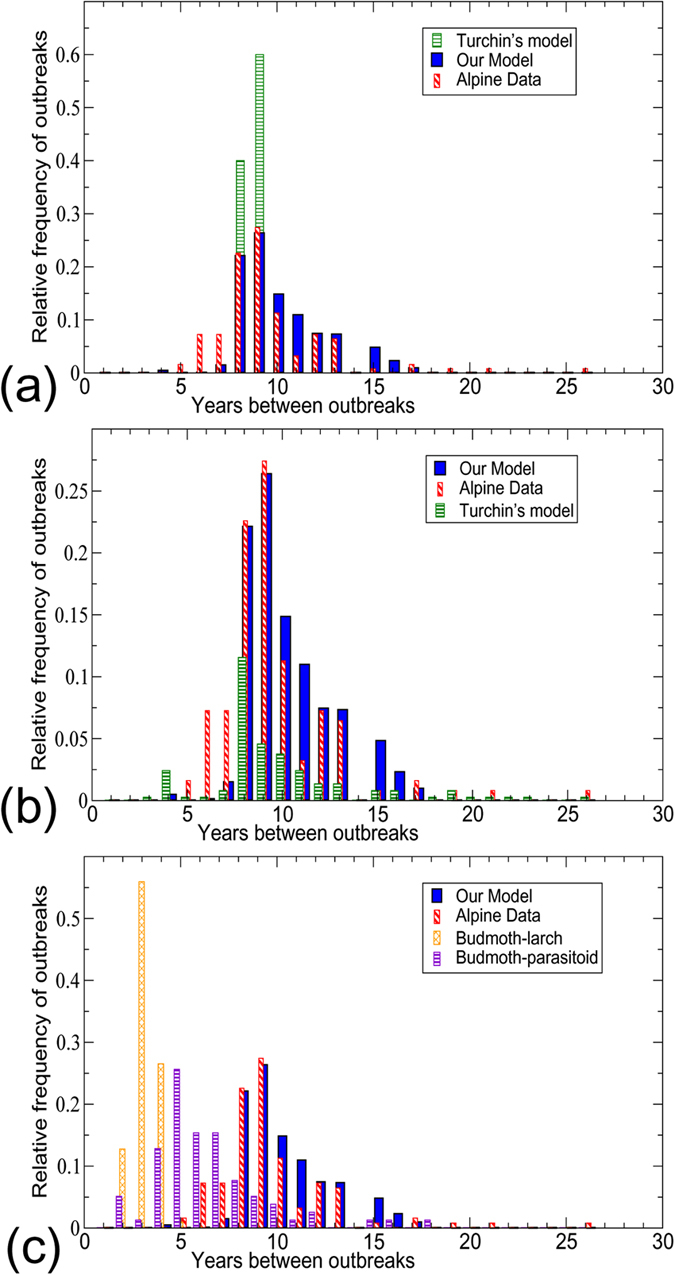
Relative frequency distributions of budmoth infestations return-time found using our model (in blue), with recorded data in ref. 1 (in red) and from Turchin’s model-2 (in (**a**)) and model-1 (in (**b**))(in green) (For Turchin’s models, his choice of parameter values: *w* = 0.17, *α* = 0.5, *k* = 250, *c* = 0.9, *γ* = 100, *δ* = 0.22, *a* = 2.5); for our model: (*q*_*y*_ = 1.13, *q*_*z*_ = 1.34, *α* = 0.5, *c* = 12, *m* = 13; *h* & *s* are varied from 0–1, and *λ* from 0–12). (**c**) 2-d *q*-deformed models comprising of either just budmoth-larch, or budmoth-parasitoid, do not yield the observed dominant cycle of 9-years. On the other hand, our model clearly captures this periodicity, explicitly validating the requirement of a deformed, tritrophic system. Differences of our model results from the Alpine data could indicate parameter regimes yet to be realised or observed.

Histograms of the frequencies of occurrence of the budmoth population cycles from our model and those generated from the budmoth outbreak data available from^1^ are very similar. In Fig. 2 our model is compared with the observed outbreaks reconstructed from tree rings data for a time-span of 1200 years^1^. Both histograms show the 9-year cycle as the predominant one followed by 8-year cycle and then a 10-year cycle. Even the relative frequencies are comparable. In ref. 1, a twenty five year cycle was assumed as no outbreak had occurred until then since 1981, and global warming (i.e., bad climatic conditions) was hypothesized as a possible reason for non-occurrence or changed periodicity of the budmoth cycles. However, no outbreak has been reported yet after 1981. Our model predicts that longer periodicities of 40 and 100 year cycles are possible (see Methods). It would be interesting therefore to check for outbreaks 5 years from now (if the 40 year cycle is realized) or if it occurs in 2081 (hence confirming the 100 year cycle). Our model successfully simulates the conditions for both the Alps as well as for Tatra and it could be used to study the system elsewhere in the world too, where the larch is endemic.

Studies indicate that there were regime shifts in the 1980s in environmental and ecological indicators driven by rapid global warming^21^ causing a global shift of the climatic system to a warmer state in a time span of only a few years. Clear warming trend of the troposphere temperatures but cooler stratosphere temperatures above Switzerland since the 1980s have been noted^22^. This^21^,^22^ appears to suggest that a climatic tipping point was reached in the 1980s, with the climatic system now being in a different (stable) state.

Environmental changes following a climatic tipping point may affect the atmosphere, hydrosphere, biosphere, etc., which in turn are reflected in various ways such as changes in the home range of animals, changes in flowering and sprouting times, changes in population cycles, etc. These changed conditions would be manifested as different phenomena in the resulting bifurcation diagrams with respect to the climate parameters. In particular, certain regions of the parameter space would act as repellers while some other regions would act as attractors, respectively creating and annihilating fixed points, changing the periodicity of the system, and taking it to a different state. The new stable equilibrium points would correspond to the new state where the system would now live in after attaining the climatic tipping point, different from the previous state of the system.

**Figure 3:**
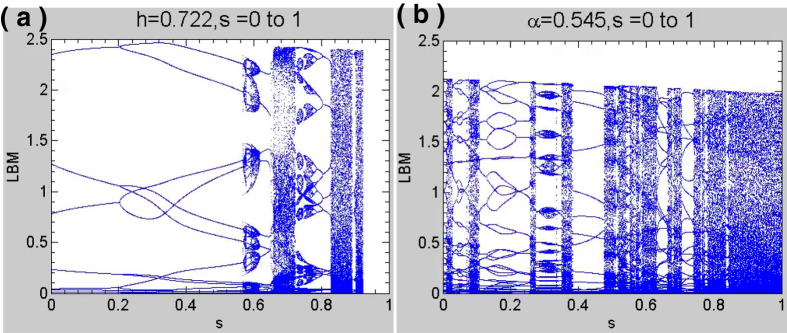
An example of (**a**) attracting and (**b**) repelling regions in the bifurcation diagrams in the Supplementary material movies, which suggest the presence of tipping points.

Some of our results depicted through movies of bifurcation diagrams for the climate parameters *h* and *s* can be viewed in the Supplementary multimedia files linked to Fig. 3. As the movies run, varied behaviour is seen: source and sink-like regions (attractors/repellors) are formed, bubble creation and destruction at specific *s* values, different chaotic bands, etc., converging from either side and merging, or getting repelled away from source. These appear to be stability boundaries of basins in the phase space of the system, demarcating regions of differing stability. This suggests that source or sink-like regions seen in the bifurcation movies may be identified with climatic tipping points.

## Conclusions

By incorporating for the first time climate effects in an ecological model bearing memory of past events, we captured the observed consequences of climate change on pest outbreak cycles, resolving the puzzle of the occurence, absence and collapse of larch budmoth cycles. We suggest using bifurcation movies for identifying climatic tipping points; and after careful calibration of climate parameters with observed data, their use for predicting future outbreaks.

## Methods

### Tritrophic model discussed in the literature

Plant quality index (PQI) which quantifies the health of the larch is defined at any given time *t* by making the leaf quality *L*_*t*_ dimensionless in relation to the average needle length (15 mm): 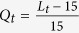. In ref. 8 the following tritrophic model (model-1) was proposed to explain population cycles of the larch budmoth.


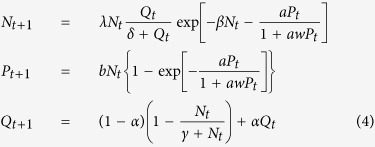


where *N*_*t*_ and *P*_*t*_ represent respectively the population densities of the budmoth and the parasitoids, *Q*_*t*_ the PQI, *β* a measure of the intra-specific competition within the budmoth population, for resources and space, *δ* the half saturation constant for the plant quality index, *γ* controls the maximum rate of the moth population uptake upon the plant, *a* the searching rate for the parasitoids, *w* the wasting time of the parasitoids; the handling time of the parasitoids has been taken to be zero in these equations^8^. The parameter *b* stands for the number of surviving parasitoids produced by each parasitized moth. Here, the parasitoid-budmoth interaction is described through the Nicholson-Bailey model^23^ except that it incorporates a different functional response (Holling type-2)^24^ for the parasitoid which mimics some of the observed data; in particular, prey consumption rises as a function of prey population density at very small prey population numbers, while plateauing to a constant value with increased prey availability. The interaction between the larch tree and the budmoth is modelled as a plant-herbivore interaction through a Ricker-like growth model^25^, and a density dependent growth rate that is proportional to the available larch needle-lengths.

In refs 7,9, a variant of this model was considered which differed only in the equation for the budmoth:


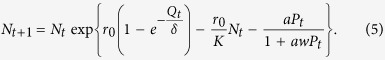


In this model (which we refer to as model-2 of Turchin), the budmoth intrinsic growth rate is dynamically generated as the system evolves. *K* is the budmoth carrying capacity and *r*_0_ is the intrinsic rate of population increase at the first time step when the system begins to evolve. The comparison histograms are plotted for both models in Fig. 2. The dynamics of Turchin’s plant-budmoth and budmoth-parasitoid models (but not the tritrophic model) were further studied in ref. 12 by rescaling to dimensionless variables:


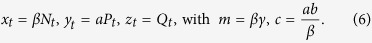


We have incorporated these transformations in our tritrophic model (equations (1, 2, 3)).

### q-deformations

A generalization of numbers and functions to *q*-deformed ones can be illustrated by observing that while the solution of the differential equation 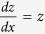 is *z* = *e*^*x*^ or *x* = ln *z*, that of 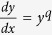 is *y* = [1 + *x*(1 − *q*)]^1/(1−*q*)^ or 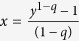. These generalized solutions 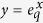 and 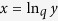 are called respectively deformed exponential and deformed logarithmic functions which give back the original exponential and logarithmic functions when *q* → 1. A deformation scheme for numbers was obtained^20^ by expanding 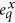 around *x* = 0:


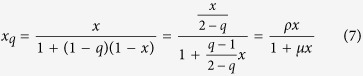


where *ρ*, *μ* are as used in Equations 1, 2, 3. These *q*-deformed numbers and functions arise naturally with Tsallis’ non-extensive statistical mechanics and preserve the Legendre transform structure of thermodynamics.

Nicholson and Bailey’s model^23^ for the budmoth-parasitoid is recovered for *q*_*y*_ = 1 (or *y*_*q*_ = *y*); this situation incorporates the assumption that the parasitoid lays eggs whenever it encounters a host – this results in overcounting of the parasitoid number. This is avoided if *q*_*y*_ ≠ 1.

Comparing Equations (1, 2, 3) with (4) enables the identification 
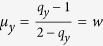
, and 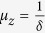, 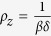 or *q*_*z*_ = 1 + *β*^11^. Since the parasitoid wasting time *w* can range from 0 to ∞, *q*_*y*_ varies between 1 and 2. The budmoth intra-specific competition can vary between 0 and ∞ but the positivity of *δ* restricts *q*_*z*_ to vary between 1 and 2. Thus *q*_*y*_ is related to the budmoth intraspecific competition and *q*_*z*_ is related to the parasitoid wasting time.

Realizations of non-Gaussian^17^ and *q*-Gaussian^18^ distributions have been demonstrated in the literature.

### Stability Analysis

The climate parameters *h*, *s* and *α* vary between 0 and 1, while the deformation parameters *q*_*z*_ and *q*_*y*_ vary between 1 and 2. Our system has the following equilibria (x^*^, y^*^, z^*^): (i) the uninfested larch (0, 0, z^*^): stable state with neither budmoth nor parasitoid, where 
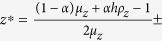
 
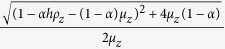
 which for *q*_*z*_ = 1 is 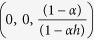, (ii) parasitoid-free states (x^*^, 0, z^*^) where 
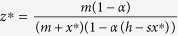
 for *q*_*z*_ = 1, and (iii) non-trivial equilibria at (x^*^, y^*^, z^*^) which simplify for *q*_*z*_ = *q*_*y*_ = 1 to 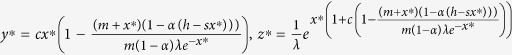 which include periodic limit cycle solutions. A linear stability analysis of the system may be done from its Jacobian which is of the form:


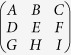


where the matrix entries are given by:


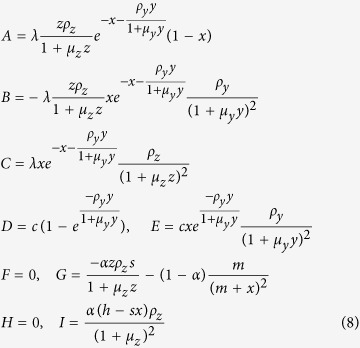


The stability of the equilibrium points may be determined using the Routh-Hurwitz criterion. For the non-trivial fixed points, the elements of the Routh array are given by:


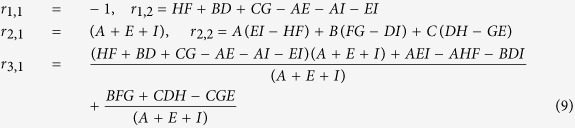



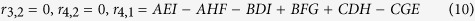


For the trivial case (uninfested larch), the Routh array simplifies to


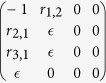



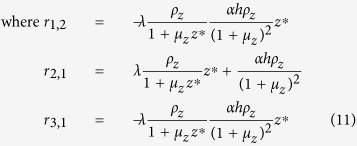


and 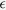 is introduced in place of zero for convenience to calculate the next entries of the array which depend upon its value. There are two sign changes in the first column of the Routh array — the trivial case is clearly unstable. Using the Routh array one also confirms that stable periodic solutions exist^26^,^27^. Stable limit cycles are generated via Neimark-Sacker bifurcations (Fig. 1).

### Histograms in Figure 2

The histograms are obtained as follows. The parameters *λ*, *h* and *s* were varied from minimum to maximum values (*h* and *s* were varied from 0 to 1 in steps of 0.1 and *λ* from 1 to 12 in steps of 0.1) to generate the time series and FFT was performed on these.

We have taken for the plant vulnerability a constant value *α* = 0.5 for the larch; this could perhaps take a different value for some other tree species. We take a constant value for *q*_*y*_ since we do not expect the parasitoid wasting time to change much unless something drastic happens to the parasitoid (such as being sprayed over by some pesticide). Similarly *q*_*z*_ related to intra-specific competition is also taken to be constant. Numerically we have checked and verified that *q*_*z*_ does not not affect the time period much.

It must be mentioned that although we have chosen the parameters to be constant, it is only under the assumption that the effect of the environment on these parameters is negligible. It could well be that there are certain thresholds beyond which these effects may not be negligible. For such scenarios one may have to additionally introduce appropriate equations governing the evolution of the parameters. We have not considered such situations for the sake of simplicity.

For Turchin’s model-2, the budmoth intrinsic growth rate is a dynamically varying quantity. However *r*_0_, the growth rate at the begining of the time step is a constant; *r*_0_ is varied from 2.3 to 2.7 as in refs 7 and 9. This corresponds to *λ* varying from 1 to 9.7336 for *r*_0_ = 2.3, to *λ* varying between 1 and 14.4593 for *r*_0_ = 2.7. Since the intrinsic growth rate is generated dynamically as *Q*_*t*_ varies from 0 to 1, several *λ* values are generated for each *r*_0_.

The simulation is performed as follows. First *r*_0_ takes a value. The system is allowed to run for 10,000 iterations. The last 1000 values are taken and FFT is performed over them, which produces the time period for these values. The maximum of the FFT is considered to be the dominant frequency and the corresponding time period is stored as an array element. If the FFT happens to be flat, the value is discarded. Sometimes the last 1000 values in the time series may not have any recurring point and hence the time period returned would be 999; this is also discarded. *r*_0_ is incremented and again a similar procedure follows. Finally an array containing, say, *N* entries of the time periods is produced. A time period of *N* ± 0.5 is assigned to Nth bin (years). The histograms are constructed by plotting the relative frequency *R*_*i*_ (defined as 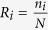 where *n*_*i*_ be the total number of occurances of N years), as a function of the years.

Since *h* and *s* of our model have not been calibrated with actual measurements from field work, we varied *h*, *s* and *λ* equally in all possible values, discarding those values of *h*, *s* and *λ* that yield no cycles, because they do not represent favourable conditions for cycles or they have not occurred, else there would have been a break in the 1200 year data.

Parameter values of *h* = 0.7, *s* = 0.84 and *λ* = 1.23, and *h* = 0.76, *s* = 0.53 and *λ* = 1.13, respectively yield 40 and 100 year cycles. These two cycles are very rare events, so that the relative frequencies of their outbreaks are both as small as about 10^−6^ and are shown in the Supplementary Information, in Fig. S4 with larger marker-size.

In conclusion, the observation that after 1981 there have been no cycles yet can mean that there have been changes in the system parameters such that either the alpine system has slipped into a non-cyclic state or it has moved into cycles with much longer time period (40, 100 years).

## Additional Information

**How to cite this article**: Iyengar, S. V. *et al*. Impact of climate change on larch budmoth cyclic outbreaks. *Sci. Rep*. **6**, 27845; doi: 10.1038/srep27845 (2016).

## Supplementary Material

Supplementary Information

Supplementary Movie S1

Supplementary Movie S2
