# Supplementary material for: Impact of climate change on larch budmoth cyclic outbreaks
Source: Sci Rep. 2016 Jun 13;6:27845. doi: 10.1038/srep27845 (PMC4904200; doi:10.1038/srep27845)
Supplement: Supplementary Information [file srep27845-s1.pdf]

Supplementary Information: Impact of climate change on larch budmoth cyclic outbreaks

Sudharsana V. Iyengar[1], Janaki Balakrishnan[2,*] and Jürgen Kurths[3]
[1]*School of Physics, University of Hyderabad, Central Univ. PO, Gachi Bowli, Hyderabad 500 046, India.*
[2]*School of Natural Sciences & Engineering, National Institute of Advanced Studies (N.I.A.S.), Indian Institute of Science Campus, Bangalore - 560012, India. and*
[3] *Potsdam Institute for Climate Impact Research, PO Box 601203, Potsdam 14412, Germany.*

Description / Legends for video files:

1. (a) For Supplementary video-1 file (SI3a_attract.mov) :
   Video of bifurcation diagram of the larch budmoth (LBM) population density x with respect to the climate parameter s for varying h.

   For low h values the bifurcation diagram seems to move towards left, i.e, shifting towards lower s values. At h=0.048 one can see the creation of an attractive region which pulls points towards it from both sides. As h increases the attractor moves towards higher values of s, though not strictly following an increasing trend. At h= 0.071, the attracting region is roughly centred around s=0.04, when h increases to 0.159, the attractor occurs near s=0.18. In the screenshot in Fig.3(a) at h=0.722, the attracting region is centred around s=0.6 and it stabilises there despite any further increase in h.

2. (b) For Supplementary video-2 file (SI3b_repel.mov) :
   Video of bifurcation diagram of the larch budmoth (LBM) population density x with respect to the climate parameter s for varying α. In the screenshot of the video (Fig.3(b)), at α=0.545 there is a repelling region centered around s=0.41. The repeller begins at higher values of s and slowly moves towards the left (decreasing s) as α increases. For instance, at α=0.23 the repelling region is formed around s=0.75; as α increases to 0.485, the repeller shifts to s=0.6, shifting further to s=0.4 when α=0.555. The repelling and attracting regions appear to be boundaries demarcating different stability regions in the phase space of the system. These sources and sinks may be identified with climatic tipping points pushing the system into different stable states.

(c) Supplementary Figure S4 showing the predicted (but rare) 40 & 100 year cycles:

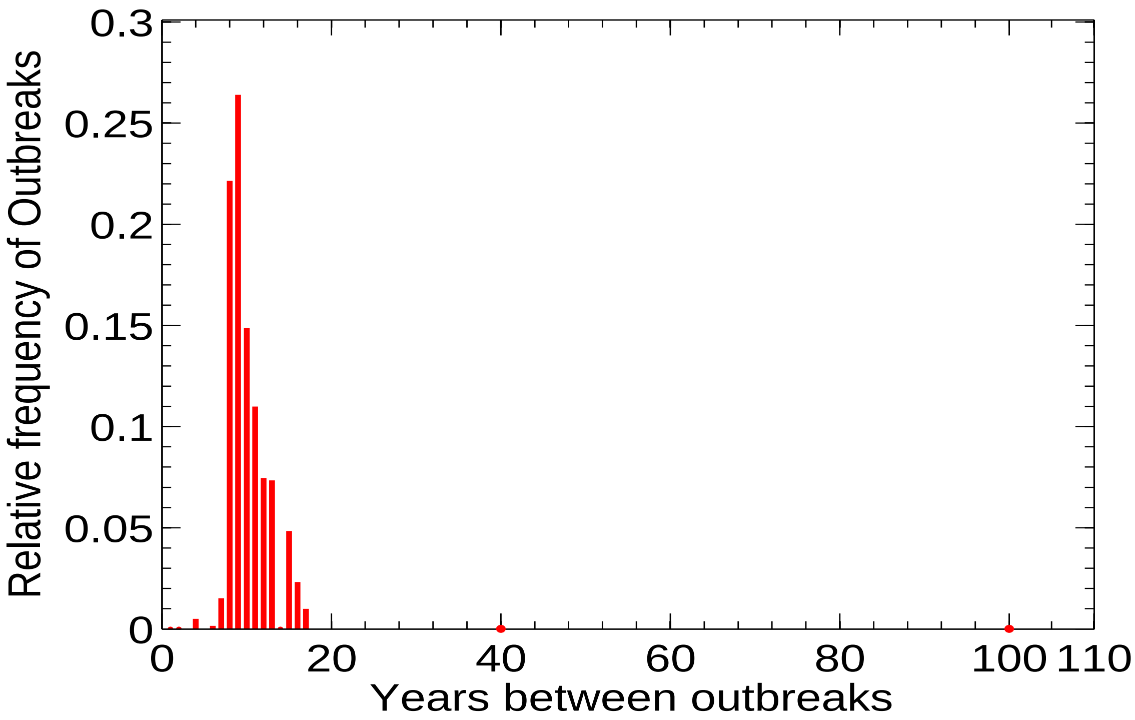

FIG. S4: Detail of the histogram showing relative frequency of budmoth outbreaks as obtained with our model, showing the predicted (though rare) 40 and 100 year cycles both depicted with increased marker-sizes for visual clarity. Parameter values of h=0.7, s=0.84 and λ=1.23, yield 40 year cycles while 100 year cycles result for h=0.76, s= 0.53 and λ=1.13.

*Corresponding author. Electronic mail: janaki05@gmail.com
